# A Parent-Delivered Foot Bath Intervention for Chemotherapy-Related Fatigue in Children with Cancer: A Family-Centered Randomized Controlled Trial

**DOI:** 10.3390/children13070921

**Published:** 2026-07-13

**Authors:** Özge Eda Karadağ Aytemiz, Şadiye Dur, Sermin Dinç

**Affiliations:** 1 Department of Pediatric Nursing, Faculty of Nursing, Koç University, 34450 Istanbul, Türkiye; 2 Department of Pediatric Nursing, Faculty of Nursing, İzmir Demokrasi University, 35140 İzmir, Türkiye; sadiye.dur@idu.edu.tr; 3 Department of Pediatric Nursing, Faculty of Health Sciences, Istanbul Atlas University, 34408 Istanbul, Türkiye; sermin.dinc@atlas.edu.tr

**Keywords:** pediatric oncology, chemotherapy-related fatigue, foot bath, family-centered care, non-pharmacological intervention, nursing

## Abstract

**Highlights:**

**What are the main findings?**

- Daily fatigue (ONS-VFS) declined significantly over 7 days in both the intervention and control groups (median 5 to 2; Friedman *p* < 0.001 for each) with no statistically significant difference between groups on any day.
- A 7-day parent-delivered, home-based foot bath was feasible and safe, with 100% adherence and no adverse events across all 61 participants.

**What is the implication of the main finding?**

- A parent-delivered foot bath is a safe, low-cost, and feasible nursing intervention that positions families as active partners in managing acute chemotherapy-related fatigue in pediatric oncology settings.
- These findings support the integration of parent-delivered, home-based non-pharmacological protocols into family-centered comprehensive symptom management plans for children receiving chemotherapy.

**Abstract:**

**Background/Objectives:** Chemotherapy-related fatigue (CRF) affects 50–80% of children receiving chemotherapy and significantly impairs quality of life. Despite the family-centered care framework guiding pediatric oncology practice, evidence on parent-delivered, home-based non-pharmacological interventions for CRF remains limited. This study aimed to evaluate the effect of a parent-delivered warm-water foot bath on CRF in children aged 7–12 years undergoing chemotherapy. **Methods:** A two-arm parallel randomized controlled trial (NCT06529484; October 2024–May 2025) was conducted at a university hospital pediatric hematology–oncology outpatient clinic. Sixty-one children with stage 3–4 Non-Hodgkin Lymphoma (intervention, *n* = 29; control, *n* = 32) were randomized using computer-generated allocation and sequentially numbered, opaque, sealed envelopes. Following structured family education, parents delivered nightly foot baths (38–40 °C, 20 min) at home for 7 consecutive days after chemotherapy administration. Adherence and fidelity were monitored via a daily WhatsApp communication protocol. Fatigue was assessed daily using the Oncology Nursing Society Visual Fatigue Scale (ONS-VFS; primary outcome) and pre/post analysis using the Pediatric Oncology Fatigue Assessment Scale (child and parent forms). Reporting followed CONSORT 2025. **Results:** Median ONS-VFS scores declined significantly over the 7 days in both groups (Friedman *p* < 0.001 for each), from 5 (Day 1) to 2 (Day 7); between-group comparisons showed no statistically significant differences on any day (all *p* > 0.05). On the Pediatric Oncology Fatigue Assessment Scale, no between-group differences favored the intervention; a single child-report subscale difference favored the control group and is most plausibly attributable to multiple comparisons. Adherence was complete (100%), no adverse events occurred, and all 61 participants completed the protocol. **Conclusions:** A parent-delivered foot bath is a safe, feasible, and well-accepted family-centered nursing intervention, with complete adherence and no adverse events. This trial was not powered to detect between-group differences and did not demonstrate superiority over standard care; adequately powered trials are needed to determine efficacy.

## 1. Introduction

Cancer is a significant cause of morbidity and mortality in childhood [1]. Chemotherapy, one of the most widely used treatment modalities, prolongs survival and improves prognosis but causes numerous adverse effects. Among these, chemotherapy-related fatigue (CRF) is the most common, significantly impacting children’s quality of life [2]. A recent systematic review and meta-analysis of 47 studies reported a pooled prevalence of fatigue in children and adolescents undergoing cancer treatment of 73%, with severe fatigue in 30% [3].

CRF negatively affects many aspects of children’s daily lives, including limitations in physical activity, sleep disturbances [4], decreased school performance, and deterioration of social relationships [5], as well as psychosocial burdens such as depression and anxiety. Critically, the impact of CRF extends beyond the child to the family system. Parents observing their child’s loss of daily functioning experience increased psychosocial burden. Within the framework of family-centered care, widely endorsed as the standard of practice in pediatric oncology, the family is recognized as the constant in the child’s life and a partner in care [6].

Pharmacological treatments are not used alone in the management of CRF; non-pharmacological methods are incorporated as supportive approaches [2]. The literature reports the use of aromatherapy, relaxation exercises, physical activity programs, massage, music therapy, and psychosocial approaches in pediatric and adult cancer patients [7,8]. Non-pharmacological methods offer the advantages of not increasing the burden of pharmacological treatment and of being amenable to nurse-led implementation. Crucially, when these interventions are designed for parents to deliver at home, they extend the reach of supportive care beyond the clinical setting, sustain therapeutic activity within daily routines, and operationalize the principles of family-centered care in concrete, replicable ways.

A foot bath is considered an easily applied nursing intervention that is low-cost and free of significant side effects. Stimulation of nerve endings in the feet by warm water can increase peripheral circulation, reduce muscle tension, and modulate the autonomic nervous system across multiple body systems [9]. The literature reports that foot baths reduce fatigue, improve sleep quality, and reduce anxiety in adult cancer patients. These characteristics, simplicity, safety, and home-based delivery, align particularly well with the family-centered care paradigm, in which the family is supported to participate actively in the child’s care [10,11,12].

Fatigue in childhood cancer is not uniform across development: appraisal, expression, and self-management of symptoms differ markedly between preschool, school-aged, and adolescent children, so interventions and outcome measures are most valid when targeted to a defined age band. The present trial, therefore, focused on school-aged children (7–12 years), for whom an age-specific, validated self-report fatigue measure is available. Although randomized controlled trials have evaluated non-pharmacological interventions for fatigue in pediatric oncology, including exercise and exergaming programs [13,14], family-system approaches that position parents as deliverers of a home-based protocol remain scarce, and warm-water foot bathing, in particular, has not been tested in this population. In this context, the present study is among the first randomized controlled trials to examine a parent-delivered foot bath for CRF in children with cancer. The study aims to contribute to the limited existing evidence and provide information on a family-centered, applicable, non-pharmacological approach to nursing care.

### Study Hypotheses

**H1.**  *Children in the intervention group will have lower post-chemotherapy ONS-VFS scores than those in the control group.*

**H2.**  *Children in the intervention group will have lower post-chemotherapy total scores on the Pediatric Oncology Fatigue Assessment Scale (Child Form) than those in the control group.*

**H3.**  *Children in the intervention group will have lower post-chemotherapy total scores on the Pediatric Oncology Fatigue Assessment Scale (Parent Form) than those in the control group.*

## 2. Materials and Methods

### 2.1. Study Design

This study used a two-arm parallel randomized controlled experimental design with a 1:1 allocation ratio. The research was conducted at the pediatric hematology–oncology outpatient clinic of a university hospital between October 2024 and May 2025. The study was conducted and reported in accordance with the CONSORT 2025 guidelines [15]. The trial was prospectively registered on ClinicalTrials.gov (NCT06529484).

### 2.2. Participants

A total of 61 children (intervention group = 29, control group = 32) and their parents participated in the study. All participants completed the follow-up. Children were eligible for inclusion if they were aged 7–12 years; had a diagnosis of stage 3 or 4 Non-Hodgkin Lymphoma; were receiving their first chemotherapy cycle; had an ONS-VFS score ≥ 3; were free of other chronic diseases; and had a parent who was literate in the local language and able to provide written informed consent. Children were excluded if they had a diagnosed cognitive developmental delay or learning difficulty, had undergone surgery within the past year, had compromised skin integrity or dermatological problems that prevented participation in the foot bath, or if the child or parent did not wish to participate. During the recruitment period, consecutively eligible children treated at the clinic were invited to participate; 61 met all inclusion criteria, consented, and were enrolled. Recruitment, eligibility screening, and enrollment were carried out by one of the researchers, a pediatric nurse, at the pediatric hematology–oncology outpatient clinic of a training and research university hospital.

### 2.3. Sample Size and Power Analysis

Sample size calculation was based on a previous pediatric oncology study examining a home-based non-pharmacological intervention for fatigue [16], which informed an a priori assumption of a medium effect size (Cohen’s *d* = 0.50) for the present design. Using G*Power 3.1 **(Heinrich-Heine-Universität Düsseldorf, Düsseldorf, Germany)**, with a two-tailed significance level of α = 0.05, desired power of 0.80, and assuming a medium effect size, the minimum required sample size was 26 participants per group. To account for a potential 20% attrition rate common in pediatric oncology studies, approximately 30 participants per group were planned. A total of 61 children were recruited and randomized. Post hoc power analysis based on the final sample indicated adequate statistical power (0.83).

### 2.4. Randomization and Allocation Concealment

Randomization was performed by an independent biostatistician using a computer-generated random number sequence. Allocation concealment was maintained using sequentially numbered, opaque, sealed envelopes prepared by the same independent biostatistician, who was not involved in recruitment, intervention delivery, or outcome assessment. Envelopes were opened only after written informed consent had been obtained and baseline assessments completed. Participants were randomly assigned to either the intervention group (parent-delivered foot bath) or the control group (standard care) in a 1:1 ratio. Due to the nature of the intervention, families and children could not be blinded to group allocation. However, outcome assessors were blinded during administration of the Pediatric Oncology Fatigue Assessment Scales, and data analysts were blinded to group assignment during statistical analysis.

### 2.5. Instruments

Information Form. A researcher-developed form that collects data on the child’s age, gender, weight, height, diagnosis, disease duration, treatment type, and medication use, as well as parental age, education, income, employment status, and care-related characteristics.

Oncology Nursing Society Visual Fatigue Scale (ONS-VFS). Developed by the Oncology Nursing Society, this single-item scale is self-reported by the child. It assesses fatigue severity on a 1 (not tired) to 5 (very tired) scale, providing a rapid and practical assessment of fatigue in pediatric oncology patients [10]. The primary outcome was daily child-reported ONS-VFS fatigue, recorded once daily over the 7 days; secondary outcomes were the total and subscale scores of the Pediatric Oncology Fatigue Assessment Scale (Child and Parent Forms), assessed at baseline and after the 7 days.

Pediatric Oncology Fatigue Assessment Scale for 7–12 Year Olds—Child Form. Developed and validated in Turkish by Kudubeş and Bektaş [17] for children aged 7–12 years, this 27-item, child self-report, Likert-type scale (1–5) assesses fatigue across three subscales (general problems, sleep problems, treatment-related problems). Higher scores indicate lower fatigue. Cronbach’s alpha = 0.98.

Pediatric Oncology Fatigue Assessment Scale for 7–12 Year Olds—Parent Form. A parallel parent proxy-report form, developed and validated in Turkish by Kudubeş and Bektaş [17], with an identical subscale structure and scoring. Cronbach’s alpha = 0.95. Written permission to use the Pediatric Oncology Fatigue Assessment Scale (Child and Parent Forms) and the ONS-VFS was obtained from the respective scale developers before data collection.

### 2.6. Intervention

Demographic and clinical characteristics were recorded using the Information Form. Before chemotherapy, each child’s fatigue level was assessed using the ONS-VFS; children with a score ≥ 3 were included, and this measurement was recorded as the pre-test for both groups.

Families in the intervention group received a structured family education session and video-based educational material detailing the purpose, duration, frequency, safety precautions, and expected effects of the foot bath. Each family was provided with an 8-L plastic basin (**Zimpfile, İstanbul, Türkiye**) and a digital thermometer (**Xenon Smart, İstanbul, Türkiye**). Parents were positioned as the primary deliverers of the intervention.

Under parental supervision, children in the intervention group received a foot bath every evening between 9:00 and 10:00 p.m. for 7 consecutive days, beginning the day after chemotherapy administration. Each session lasted 20 min, with the child’s feet immersed in water at 38–40 °C to a depth of approximately 18 cm, fully covering the calf muscles. Parents verified water temperature with the provided thermometer before each session. Parents completed a daily log sheet recording session adherence, water temperature, session duration, and any observations regarding tolerance. To support intervention fidelity and enable real-time monitoring of adherence and safety, daily remote monitoring was conducted via a secure WhatsApp communication channel established with each family. On each of the 7 intervention days, parents sent the researcher a brief written confirmation message reporting session completion and any observations regarding the child’s tolerance, together with a digital photograph of the foot bath setup at the start of the session, showing the thermometer reading and the basin with water at the specified depth. This dual-channel approach allowed direct visual verification of two key fidelity parameters, water temperature and immersion depth, for every session across all 7 days. It provided the researcher with immediate opportunities to respond to family questions or concerns. WhatsApp messages and photographs were stored in a password-protected research database in accordance with data protection requirements, and identifying information visible in any photograph was redacted before storage.

**Figure 1:**
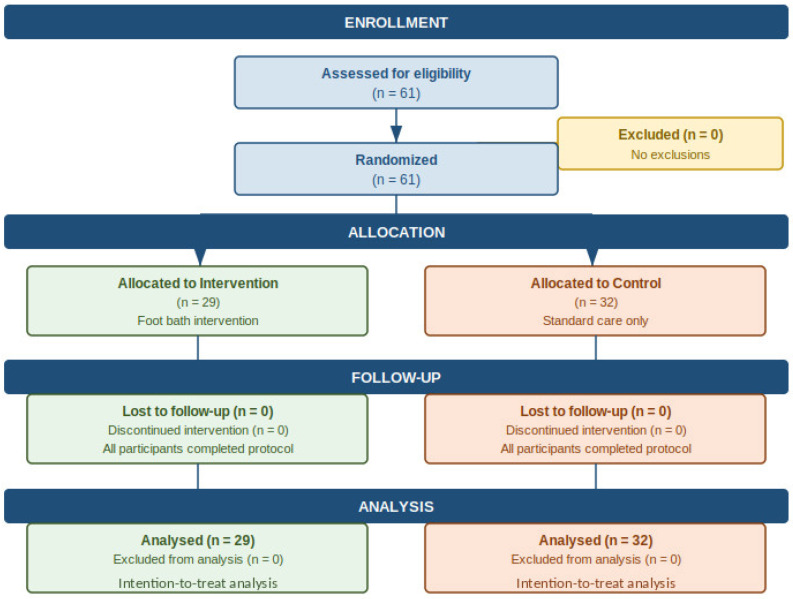
CONSORT 2025 flow diagram of participant enrollment, allocation, follow-up, and analysis. ITT = intention-to-treat.

The control group received only standard care, following the same measurement protocol without any intervention. No participants dropped out, and all completed the protocol. The CONSORT flow diagram is shown in Figure 1.

### 2.7. Statistical Analysis

Data were analyzed using SPSS for Windows, Version 26.0 (IBM Corp., Armonk, NY, USA). Descriptive statistics (number, percentage, mean, SD, minimum, maximum, median) were used. Normality was evaluated with the Shapiro–Wilk test. Parametric tests were used for normally distributed data; nonparametric tests were used otherwise. Within-group pre–post comparisons used paired t-tests or Wilcoxon signed-rank tests; between-group comparisons used independent-samples *t*-tests or Mann–Whitney *U* tests. For the repeated 7-day ONS-VFS measurements, the Friedman test with Bonferroni-corrected Conover post-hoc analysis was applied. Categorical variables were analyzed with chi-square or Yates-corrected chi-square tests. Statistical significance was set at *p* < 0.05. Because no participant withdrew (0% attrition), there were no missing outcome data, and the intention-to-treat and per-protocol samples were identical; no imputation was required. As baseline characteristics were comparable between groups (Table 1), no adjustment for confounding variables was performed.

## 3. Results

### 3.1. Demographic and Clinical Characteristics

The mean age of participants was 8.91 ± 1.75 years. Most participants were under 10 years of age (63.9%) and female (59.0%). No statistically significant differences were found between groups in baseline demographic or clinical characteristics *(p* > 0.05), confirming successful randomization and group homogeneity (Table 1).

### 3.2. Pediatric Oncology Fatigue Assessment Scale-Child Form

No between-group differences favored the intervention; the only statistically significant between-group difference emerged on the treatment-related problems subscale and favored the control group (post-test *p* = 0.012). On the treatment-related problems subscale, the intervention group remained stable (pre-test 10.58 ± 1.24; post-test 10.62 ± 1.32; *p* = 0.33), whereas the control group showed a small but significant within-group increase (11.03 ± 1.38 to 11.44 ± 1.13; *p* = 0.04). Because higher scores on this scale indicate lower fatigue, this change reflects a slight reduction in treatment-related fatigue in the control group rather than in the intervention group; as this was one of several subscale comparisons, it is most plausibly a chance finding (Table 2).

### 3.3. Pediatric Oncology Fatigue Assessment Scale-Parent Form

No significant differences were found in total scores or in any subscale (general problems, sleep problems, treatment-related problems) for either within-group or between-group comparisons (*p* > 0.05), consistent with the child self-report findings (Table 3).

### 3.4. Daily ONS-VFS Scores

A significant, progressive decline in fatigue scores was observed over the 7 days in both groups (Friedman *p* < 0.001 for each). Median scores declined from 5 on Day 1 to 2 on Day 7 in the intervention group, with a comparable decline in the control group. Post hoc Bonferroni-corrected Conover analysis confirmed that, within each group, Day 1 scores were significantly higher than those on all subsequent days. However, between-group comparisons showed no statistically significant difference on any of the 7 days (Mann–Whitney U, all *p* > 0.05; Table 4). No adverse events were observed in either group.

## 4. Discussion

Chemotherapy-related fatigue is a highly prevalent and distressing symptom in pediatric oncology for which simple, low-risk, parent-deliverable non-pharmacological options remain scarce; this trial was designed to test whether a home-based, parent-delivered warm-water foot bath could reduce acute fatigue in school-aged children while operationalizing family-centered care. This randomized controlled trial found that daily fatigue (ONS-VFS) declined significantly over 7 days in both the intervention and control groups, with no statistically significant difference between groups. The parent-delivered foot bath was feasible and safe, achieving complete adherence and no adverse events. Within this single-cycle, 7-day design, the intervention did not produce a detectable reduction in fatigue beyond that observed with standard care; however, the trial was not powered to detect between-group differences.

These findings align with existing evidence in adult oncology populations. Yang et al. [10] and Akyuz Ozdemir and Can [11] reported significant reductions in fatigue after warm-water foot baths in gynaecologic and oncology patients receiving chemotherapy. Uysal and Topbaş [18] found in their meta-analysis of 11 studies that foot baths reduced fatigue, with a moderate effect size (SMD = −0.98, *p* = 0.03). The present study extends this evidence to a pediatric population while introducing a parent-delivered, home-based intervention that operationalizes family-centered care.

Warm-water immersion has been documented to exert multi-system effects, including improved peripheral circulation, reduced muscle tension, and parasympathetic activation [9]. Yamamoto and Nagata [12] demonstrated that warm footbaths reduce sympathetic activity, increase salivary immunoglobulin levels, and lower cortisol responses in hospitalized patients with cancer, reflecting reduced physiological stress and improved immune function.

Foot baths demonstrate comparable or superior feasibility relative to other evidence-based non-pharmacological interventions for cancer-related fatigue and pain. A meta-analysis of randomized trials on massage therapy in adult cancer pain populations reported variable effects across studies, with limited evidence of robust functional improvement [19]. Inhalation aromatherapy in cancer patients has shown mixed results, with no significant effect on pain or depression and only modest effects on anxiety and sleep [20]. In children and adolescents with cancer, a systematic review of non-pharmacological interventions for fatigue reported that six of the nine included studies showed statistically significant reductions in fatigue. Still, heterogeneity across interventions was substantial [5]. Direct head-to-head comparisons are needed to establish relative effectiveness.

The 7–12-year age band, although covered by a single validated instrument, still spans a broad developmental range. Younger school-aged children (7–8 years) typically depend more on caregivers for symptom appraisal, emotional regulation, and adherence, whereas older children (10–12 years) show greater autonomy, self-awareness, and capacity for self-management. These differences may shape both how fatigue is experienced and reported and how children engage with a parent-delivered intervention, and could contribute to the within-group variability that a modest sample cannot fully resolve. Future trials should stratify or analyze outcomes by developmental stage and consider age-adapted delivery, so that intervention intensity and the balance of parental versus child involvement match the child’s developmental capacity.

A further consideration is the child’s active role. Because the protocol positioned parents as the primary deliverers of care, the design may appear to cast children as passive recipients. In practice, children were not passive: they self-administered the foot bath under supervision, could decline or stop a session at any time, and their daily self-reported fatigue directly informed the primary outcome. Recognizing and strengthening this agency, by inviting children to co-set routines, express preferences, and appraise their own symptoms, is consistent with child- and family-centered care and may enhance engagement and the therapeutic value of home-based interventions. Future studies could incorporate child-reported measures of acceptability and participation to capture this dimension better.

### 4.1. Implications for Pediatric Nursing Practice

The evidence-based parameters established in this study, water temperature 38–40 °C, session duration 20 min, administered daily during chemotherapy cycles, translate directly into clinical practice. The intervention requires minimal equipment (a basin, thermometer, and clean water), making it feasible in both institutional and home settings. Nurses should provide structured family education, written protocols with safety precautions, and coordinate with the multidisciplinary team regarding patient eligibility. Critically, nurses are positioned to lead the implementation of family-centered care by designing and disseminating parent-delivered protocols.

### 4.2. Limitations

This study has several limitations. First, generalizability may be limited as the sample comprised only children with Non-Hodgkin Lymphoma; fatigue patterns and intervention responses may differ in other pediatric cancer types. Second, only a single intervention dose was evaluated; optimal duration, temperature, and frequency remain undetermined. Third, the 7-day follow-up captures only acute effects; sustained benefits beyond the immediate chemotherapy cycle are unknown. Fourth, physiological mechanisms (cortisol, inflammatory markers, autonomic nervous system activity) were not directly measured. Fifth, the single-center design limits generalizability. Sixth, because of the nature of the intervention, families and children could not be blinded, introducing the possibility of expectancy bias in this subjective outcome. Seventh, although sex was reported as a descriptive variable, sex- and gender-based analyses were not conducted; future studies should include them. Eighth, although the sample met the a priori power calculation for the primary outcome, the overall sample size (N = 61) was modest, limiting power to detect small between-group differences and precluding subgroup analyses. Future studies should also consider active control designs (e.g., room-temperature foot soaking) to isolate treatment-specific effects, and incorporate parent-reported outcomes that more fully capture the family-centered dimensions of intervention impact.

## 5. Conclusions

This study provides preliminary evidence that a parent-delivered foot bath is a safe, feasible, and well-accepted family-centered nursing intervention for children with cancer aged 7–12 years, achieving complete adherence and no adverse events. Although daily fatigue declined significantly within both groups, no between-group benefit over standard care was detected, and the trial was not powered to confirm efficacy. Adequately powered, multicenter trials are needed before foot bathing can be recommended for routine inclusion in symptom-management protocols. Future research should examine parent-delivered foot bathing combined with other non-pharmacological interventions; optimal timing relative to chemotherapy administration; the long-term sustainability of home-based implementation; applicability across diverse pediatric cancer diagnoses; and the broader effects of family-delivered protocols on parental caregiving experience and family well-being. For nursing practice, the intervention’s safety, simplicity, and full acceptability support offering parent-delivered foot bathing as an optional, family-engaging comfort measure within routine supportive care, while its specific contribution to fatigue reduction awaits confirmation in adequately powered trials.

## Figures and Tables

**Table 1:** Demographic and clinical characteristics of participants (N = 61).

Characteristic	Intervention (n = 29)	Control (n = 32)	Total (N = 61)	*p*
Age (years), Mean ± SD	8.69 ± 1.71	9.10 ± 1.79	8.91 ± 1.75	0.337 ^a^
Age, Min–Max	7–12	7–12	7–12	-
Age < 10 years, n (%)	20 (69.0)	19 (59.4)	39 (63.9)	0.609 ^b^
Age ≥ 10 years, n (%)	9 (31.0)	13 (40.6)	22 (36.1)	-
Female, n (%)	15 (51.7)	21 (65.6)	36 (59.0)	0.400 ^b^
Male, n (%)	14 (48.3)	11 (34.4)	25 (41.0)	-

SD: standard deviation. ^a^ Independent-samples *t*-test. ^b^ Yates-corrected chi-square test.

**Table 2:** Pre-test and post-test scores on the Pediatric Oncology Fatigue Assessment Scale (Child Form) by group.

Subscale/Time	Intervention (n = 29)	Control (n = 32)	Total (N = 61)	t	*p*
General problems					
Pre-test	68.85 ± 5.39	70.07 ± 4.71	69.50 ± 5.03	−0.905	0.370
Post-test	68.55 ± 5.64	69.97 ± 5.57	69.30 ± 5.60	−0.987	0.328
Difference	0.12 ± 1.31	0.13 ± 3.95	0.13 ± 3.00	−0.022	0.982
Within-group	t = −0.450; *p* = 0.656	t = −0.185; *p* = 0.855	t = −0.311; *p* = 0.757	-	-
Sleep problems					
Pre-test	22.08 ± 1.92	22.27 ± 2.27	22.18 ± 2.10	−0.335	0.739
Post-test	22.14 ± 1.81	22.63 ± 2.09	22.39 ± 1.96	−0.969	0.337
Difference	0.08 ± 1.47	0.43 ± 2.27	0.27 ± 1.93	−0.686	0.496
Within-group	t = −0.267; *p* = 0.791	t = −1.046; *p* = 0.304	t = −1.038; *p* = 0.304	-	-
Treatment-related problems					
Pre-test	10.58 ± 1.24	11.03 ± 1.38	10.82 ± 1.32	−1.296	0.201
Post-test	10.62 ± 1.32	11.44 ± 1.13	11.05 ± 1.28	−2.598	0.012 *
Difference	−0.04 ± 0.20	0.37 ± 0.93	0.18 ± 0.72	−2.182	0.033 *
Within-group	t = 1.000; *p* = 0.327	t = −2.164; *p* = 0.039 *	t = −1.866; *p* = 0.067	-	-
Total score					
Pre-test	101.50 ± 6.27	103.37 ± 5.66	102.50 ± 5.97	−1.171	0.247
Post-test	101.31 ± 6.74	104.03 ± 7.12	102.74 ± 7.02	−1.528	0.132
Difference	0.15 ± 2.05	0.93 ± 4.96	0.57 ± 3.88	−0.747	0.458
Within-group	t = −0.382; *p* = 0.706	t = −1.030; *p* = 0.311	t = −1.102; *p* = 0.275	-	-

SD: standard deviation. Between-group comparisons: independent-samples *t*-test; within-group comparisons: paired-samples *t*-test. * *p* < 0.05.

**Table 3:** Pre-test and post-test scores on the Pediatric Oncology Fatigue Assessment Scale (Parent Form) by group.

Subscale/Time	Intervention (n = 29)	Control (n = 32)	Total (N = 61)	T	*p*
General problems					
Pre-test	68.96 ± 4.09	69.43 ± 4.61	69.21 ± 4.34	−0.402	0.689
Post-test	68.69 ± 5.43	69.81 ± 5.35	69.28 ± 5.37	−0.813	0.420
Difference	0.12 ± 6.13	0.57 ± 4.17	0.36 ± 5.13	−0.326	0.746
Within-group	t = −0.096; *p* = 0.924	t = −0.744; *p* = 0.463	t = −0.521; *p* = 0.604	-	-
Sleep problems					
Pre-test	23.04 ± 1.87	22.27 ± 2.23	22.63 ± 2.09	1.393	0.169
Post-test	22.48 ± 2.01	22.53 ± 2.23	22.51 ± 2.11	−0.089	0.929
Difference	−0.50 ± 1.77	0.33 ± 1.92	−0.05 ± 1.88	−1.680	0.099
Within-group	t = 1.439; *p* = 0.163	t = −0.952; *p* = 0.349	t = 0.213; *p* = 0.832	-	-
Treatment-related problems					
Pre-test	10.85 ± 0.97	11.13 ± 1.31	11.00 ± 1.16	−0.923	0.360
Post-test	10.76 ± 1.24	11.34 ± 1.36	11.07 ± 1.33	−1.748	0.086
Difference	−0.15 ± 1.32	0.17 ± 1.34	0.02 ± 1.33	−0.899	0.373
Within-group	t = 0.595; *p* = 0.557	t = −0.681; *p* = 0.502	t = −0.101; *p* = 0.920	-	-
Total score					
Pre-test	102.85 ± 4.82	102.83 ± 6.68	102.84 ± 5.84	0.008	0.994
Post-test	101.93 ± 6.41	103.69 ± 7.07	102.85 ± 6.77	−1.013	0.315
Difference	−0.54 ± 7.03	1.07 ± 4.81	0.32 ± 5.94	−1.009	0.318
Within-group	t = 0.391; *p* = 0.699	t = −1.216; *p* = 0.234	t = −0.405; *p* = 0.687	-	-

SD: standard deviation. Between-group comparisons: independent-samples *t*-test; within-group comparisons: paired-samples *t*-test.

**Table 4:** Daily fatigue scores were assessed by the Oncology Nursing Society Visual Fatigue Scale over 7 days.

Day	Intervention (n = 29) Median (IQR)	Control (n = 32) Median (IQR)	Total (N = 61) Median (IQR)	U	*p*
Day 1	5 (5–5)	5 (4–5)	5 (4–5)	549.000	0.123
Day 2	4 (4–4)	4 (3–4)	4 (3–4)	430.000	0.572
Day 3	4 (3–5)	4 (4–4)	4 (3–4)	536.500	0.212
Day 4	3 (3–4)	4 (3–4)	4 (3–4)	456.500	0.904
Day 5	3 (3–3)	2 (2–3)	3 (2–3)	363.000	0.091
Day 6	2 (2–2)	2 (2–3)	2 (2–3)	462.000	0.973
Day 7	2 (2–2)	2 (2–3)	2 (2–3)	387.500	0.197
Kendall’s W	W = 0.687; *p* < 0.001	W = 0.770; *p* < 0.001	W = 0.727; *p* < 0.001	Friedman	

IQR: interquartile range (Q1–Q3). Between-group comparisons: Mann–Whitney U test; within-group comparisons over the 7 days: Friedman test.

## Data Availability

The datasets generated and analyzed in the current study are not publicly available due to privacy and ethical restrictions, but are available from the corresponding author upon reasonable request.
